# Molecular detection of flaviviruses and alphaviruses in mosquitoes (Diptera: Culicidae) from coastal ecosystems in the Colombian Caribbean

**DOI:** 10.1590/0074-02760160096

**Published:** 2016-10-03

**Authors:** Richard Hoyos-López, Juan Suaza-Vasco, Guillermo Rúa-Uribe, Sandra Uribe, Juan Carlos Gallego-Gómez

**Affiliations:** 1 Universidad de Antioquia, Translational and Molecular Medicine Group, Medellín, Antioquia, Colombia; 2 Universidad Nacional de Colombia, Grupo de Investigación en Sistemática Molecular, Medellín, Antioquia, Colombia; 3 Universidad de Antioquia, Facultad de Medicina, Grupo de Entomología Médica, Medellín, Antioquia, Colombia

**Keywords:** alphavirus, flavivirus, mosquitoes, mangroves, emerging infectious diseases, molecular detection

## Abstract

Arboviruses belonging to the genera *Flavivirus* and *Alphavirus* were detected in mosquitoes in a rural area of San Bernardo del Viento (Córdoba, Colombia). A total of 22,180 mosquitoes were collected, sorted into 2,102 pools, and tested by generic/nested reverse transcription-polymerase chain reaction. Venezuelan equine encephalitis virus, dengue virus, West Nile virus, St. Louis encephalitis virus, yellow fever virus, and *Culex flavivirus* were detected and identified by sequencing. The detection of arboviral pathogens in this zone represents possible circulation and indicates a human health risk, demonstrating the importance of virological surveillance activities.

The known mosquito-associated arboviruses in Colombia are found in three families and four genera of medically important viruses: *Flaviviridae* (*Flavivirus*), *Togaviridae* (*Alphavirus*), and *Bunyaviridae* (*Orthobunyavirus* and *Phlebovirus*) (Groot 1996, Weaver 2005). Representative pathogenic arboviruses associated with human illness in South America include the West Nile virus (WNV), dengue virus (DENV), Venezuelan equine encephalitis virus (VEEV), eastern equine encephalitis virus (EEEV), Saint Louis encephalitis virus (SLEV), yellow fever virus (YFV), Zika virus (ZIKV), and Chikungunya virus (CHIKV) (Gubler 2008, Weaver & Reisen 2010). Negeviruses represent another important group. These recently described viruses comprise insect-specific viruses found in mosquitoes and phlebotomine sandflies (Vasilakis et al. 2013, Auguste et al. 2014, Nunes et al. 2015).

There are historical reports of alphaviruses (VEEV, EEEV, Mayaro), flaviviruses (YFV, WNV, DENV, SLEV, Ilheus, Bussuquara), and bunyaviruses (*Anopheles* A, *Anopheles* B, Guaroa, Oya, Oropouche, Wyeomyia) in areas of the Magdalena river valley, Llanos Orientales, Gulf of Uraba, Guajira, Catatumbo, Caribbean, and Pacific coast of Colombia (Groot 1964, Prías-Landínez et al. 1968, SanMartín et al. 1971, Rivas et al. 1995, Weaver et al. 1996, 2004, Hastriter & Lawyer 1998, Ferro et al. 2003, 2008, Méndez et al. 2003, Jaramillo et al. 2005, Mattar et al. 2005a, b, 2011, Álvarez et al. 2010, Góez-Rivillas et al. 2010, Múnera et al. 2010, Osorio et al. 2012, Barrera et al. 2015, Hoyos-López et al. 2015a, b, c). Moreover, mosquitoes in the genera *Aedes*, *Anopheles*, and *Culex* (including subgenera *Culex* and *Melanoconion*), and *Haemagogus*, and in the tribe Sabethini are recognised as potential vectors of such viruses (Roca-Garcia 1944, Groot 1964, Ferro et al. 2003, 2008, Jaramillo et al. 2005). Continuous ecological change, including fragmentation of natural ecosystems, may increase the probability of human-vector contact (Hastriter & Lawyer 1998, Ferro et al. 2015) and, consequently, the possibility of outbreaks of emerging and re-emerging arboviruses. Although some studies have focused on arbovirus detection in wild mosquito populations in Colombia, these reports are relatively scarce. This is despite efforts in the last few years to record potential vector mosquito species, to identify the vector species in recent arboviral outbreaks, and to validate molecular techniques for viral detection (Ferro et al. 2003, 2008, González-Reiche 2010, Parra-Henao & Suárez 2012, Barajas et al. 2013, Hoyos-López et al. 2015a, b, c).

The present study provides an update on emerging and re-emerging arboviruses in Colombia, particularly for the Caribbean coastal zone and for flaviviruses and alphaviruses. Results were obtained as a part of a multidisciplinary study conducted between 2011-2013 in San Bernardo del Viento (Córdoba department, Colombia), an area poorly studied in terms of arboviruses, despite exhibiting particular conditions such as the presence of migratory birds and other fauna that might act as viral reservoirs and recent anthropogenic activities (fragmentation of mangrove forests, expansion of rice fields, and expansion of cattle ranching) that might promote the transmission of arboviruses to humans.

## MATERIALS AND METHODS

**Fig. 1:**
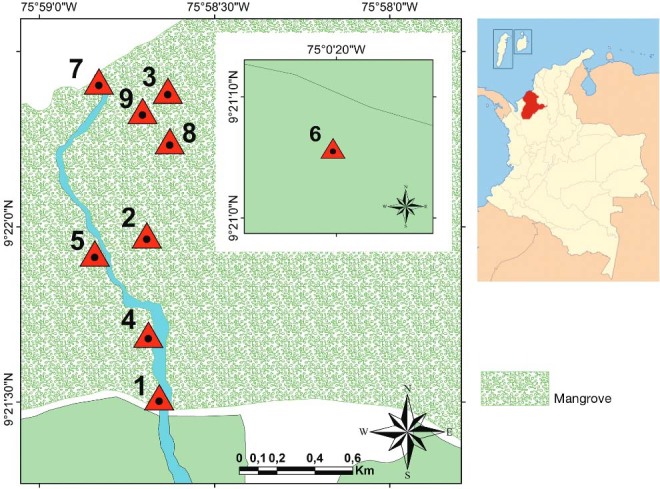
: map of the study area in San Bernardo del Viento (Córdoba). Precise sampling locations: (1) human domiciles in “La Balsa”; (4) pastures; (2) rice fields in mangrove forest; (3), (5), (7), (9), (8) mangrove forests; (6) secondary forest. The distance between the secondary forest and the rural zone “La Balsa” is 7.36 km.

*Study area and mosquito samples* - Mosquitoes were collected at eight sites in a rural area called “La Balsa” (9º 21´ 30.97” N, 75º 58´ 37.28” W) in San Bernardo del Viento (Fig. 1). San Bernardo del Viento (approximate population: 32,000) is two meters above sea level, has a mean annual temperature of 30ºC and is bordered by the Sinú River delta. The study area consisted of numerous mangrove trees (across 74 ha), such as “Mangle rojo” (*Rhizophora mangle*), “Mangle bobo” (*Laguncularia racemosa*), “Zaragoza” (*Conocarpus erectus*), “Mangle negro” (*Avicennia germinans*), and “Mangle piñuelo” (*Pelliciera rhizophorae*), with a canopy averaging 24 m in height (Rojas & Sierra-Correa 2010). The ground is well irrigated by streams transecting the study site that eventually form ponds and ditches. Small rice fields cultivated by local residents near their homes and inside the mangrove habitats were also inspected. Cattle, chickens, goats, horses, and donkeys were the most common peridomestic animals observed in farm areas close (within approximately 400 m) to the mangroves. Mosquitoes, namely resting and engorged females, were manually sampled.

**Fig. 2:**
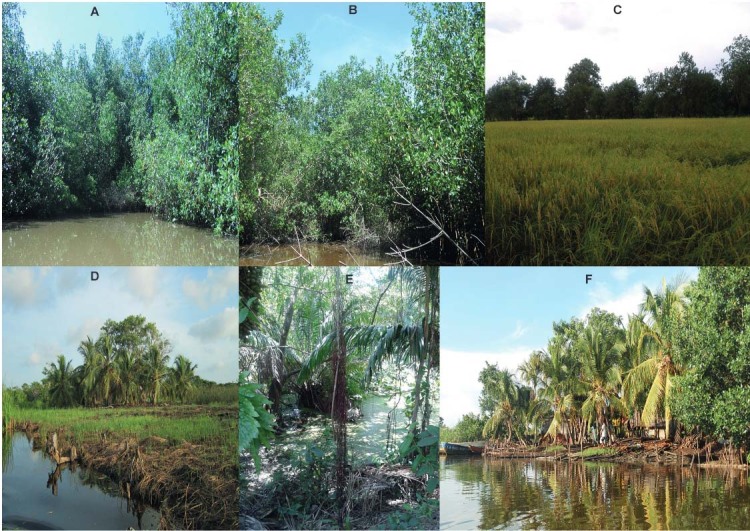
: ecosystems sampled in San Bernardo del Viento (Córdoba). (A-B) Mangrove forest; (C) rice fields in mangrove forest; (D) pastures; (E) secondary forest; (F) houses around mangroves (approximately 500 m).

Adult mosquitoes were collected on seven field trips between September 2011 and October 2013, during seasons (February-April, September-November) of peak bird migration. Each field trip consisted of five to seven days of sampling. Mosquitoes were collected using eight dry ice-baited CDC light traps operated for a 14 h period from 5:00 p.m. to 7:00 a.m. The light traps were placed in the mangrove forest, in small rice fields inside this forest, at the edge of mangroves, in secondary forests, and in peridomestic sites (Fig. 2). Additionally, manual and electric aspirators were used during the day (9:00 a.m.-4:00 p.m.) to collect mosquitoes in their resting sites.

After each collection, mosquitoes were sorted and pooled in the field using a cold table. Mosquitoes were pooled based on similar external morphological characteristics. Samples were stored in cryovials containing 1-50 specimens, placed in a liquid nitrogen tank, and transported to the insectary (Universidad Nacional de Colombia, Medellín). A reference mosquito collection that includes morphospecies identified during the field trips was established. Mosquitoes were mounted and identified using morphological keys (Lane & Cerqueira 1942, Lane 1953, Cova-Garcia 1966, Bram 1967, Arnell 1973, Berlin & Belkin 1980, Sirivanakarn 1982, Clark-Gil & Darsie Jr 1983, Pecor et al. 1992, Forattini 2002a, González & Carrejo 2007). In addition, a barcoding methodology was used to identify mosquitoes (Kumar et al 2007, Hoyos-López et al. 2015a, b, c, d). Protocols were implemented as described in the International Barcode of Life (iBOL) project (http://www.ibol.org/).

*Arbovirus detection by reverse-transcription polymerase chain reaction (RT-PCR) and sequencing* - Each mosquito pool tested for viruses was triturated in a cold mortar with sterile minimum essential medium (MEM) supplemented with 10% foetal bovine serum and 1% penicillin and then clarified by centrifugation at 13000 rpm for 30 min. The supernatant was poured into Eppendorf tubes, and total RNA was extracted using an RNeasy Kit (Qiagen, Valencia, CA, USA). Generic (RT-PCR) and nested (RT-nested PCR) were performed using a One-Step RT-PCR kit (Qiagen, Valencia). The protocols used for flavivirus and alphavirus detection were those described by Sánchez-Seco et al. (2001, 2005) (Supplementary data, Table). The viruses used as positive controls were DENV (New Guinea strain) and VEEV (Argentina strain) donated by Dr Luis Adrián Díaz (Universidad Nacional de Córdoba). Positive samples were sequenced in both directions using an ABI automatic sequencer (Macrogen, Korea), and the resulting sequences were compared with GenBank reference sequences.

*Sequence and data analyses* - Nucleotide sequences of positive samples were compared with those of reference strains from each viral group (Sánchez-Seco et al. 2001, 2005). Sequences obtained from the first product amplified by RT-PCR (Sánchez-Seco et al. 2005) were aligned using Bioedit v7.0 (Hall 1999). The neighbour-joining algorithm (Saitou & Nei 1987) and Kimura 2-parameter model (Nei & Kumar 2000) were used to construct a dendrogram in MEGA v6.0 software (Tamura et al. 2013). Sequences were compared with those available from GenBank using BLASTN (Altschul et al. 1997). Minimum infection rates (MIRs) were calculated as follows: MIR = number of positive pools by morphospecies/total number of morphospecies tested from that site × 1000 (Walter et al. 1980).

*Ethics* - Mosquitoes were collected according to the guidelines of Colombian decree number 1376 (2013), which regulates the collection of wild specimens of biological diversity for non-commercial research. No specific permits were required for this study.

## RESULTS

A total of 2102 pools, representing 14 morphospecies, were screened. Mosquitoes were identified based on morphology and DNA barcoding. A total of 268 voucher specimens of mosquito species were deposited in the Francisco Luis Gallego Entomological Museum.

Sequences from alphaviruses and flaviviruses were amplified from 30 of the 2102 mosquito pools (1.43%) (Table). Mosquitoes belonging to the genus *Culex* according to morphology were collected most frequently. *Deinocerites* (*De. atlanticus* aff.), which were present in mangrove forests with numerous crab holes, were also common.

**TABLE:** Morphospecies, pools tested, viruses detected, and minimum infection rates (MIRs) by mosquito species

Mosquito species	Mosquito tested	Pools tested	Arboviruses identified in positive pools	MIR*
*Culex* spp.	10011	816	VEEV (8) SLEV (1) WNV (2) CxFv (2)	0.799 0.099 0.199 0.199
*Deinocerites atlanticus* aff.	6015	582	VEEV (2)	0.332
*Mansonia titillans*	1887	259	VEEV (3) SLEV (1)	1.59 0.529
*Aedes* (*Stegomyia*) *aegypti*	1150	107	DENV2 (4)	3.47
*Culex* (*Culex*) *quinquefasciatus*	1019	99	CxFv (4)	3.92
*Haemagogus* sp. (Splendens section)	757	78	YFV (1)	1.32
*Psorophora* (*Grabhamia*) *confinnis*	622	71	VEEV (1)	1.61
*Aedes scapularis*	512	62	VEEV (1)	1.95
*Aedeomyia squamipennis*	83	11	-	-
*Ochlerotatus taeniorhynchus*	72	9	-	-
*Anopheles aquasalis*	35	5	-	-
*Anopheles neomaculipalpus*	17	3	-	-

Total	22180	2102	6 (30)	-

*: MIR per 1000 mosquitoes. VEEV (Venezuelan equine encephalitis virus); SLEV (St. Louis encephalitis virus); WNV (West Nile virus); CxFV (*Culex flavivirus*); YFV (yellow fever virus); and DENV (dengue virus).

DNA barcodes were obtained for mosquitoes in the genus *Culex*; however, closely related species within the subgenera *Culex* and *Melanoconion* could not be distinguished by their sequences, and an exact species could not be assigned to specimens other than *Cx*. (*Culex*) *quinquefasciatus* (KT766432–KT766453).

Nested RT-PCR was used to identify viruses in mosquito pools. However, generic RT-PCR with primers targeting flaviviruses (1.1 kb, NS5 gene) and alphaviruses (500 b, nsP4 gene) were also obtained and sequenced, for consensus alignments of 515 and 420 nt, respectively. These sequences were used for identification by BLASTN search and neighbour-joining dendrograms.

**Fig. 3:**
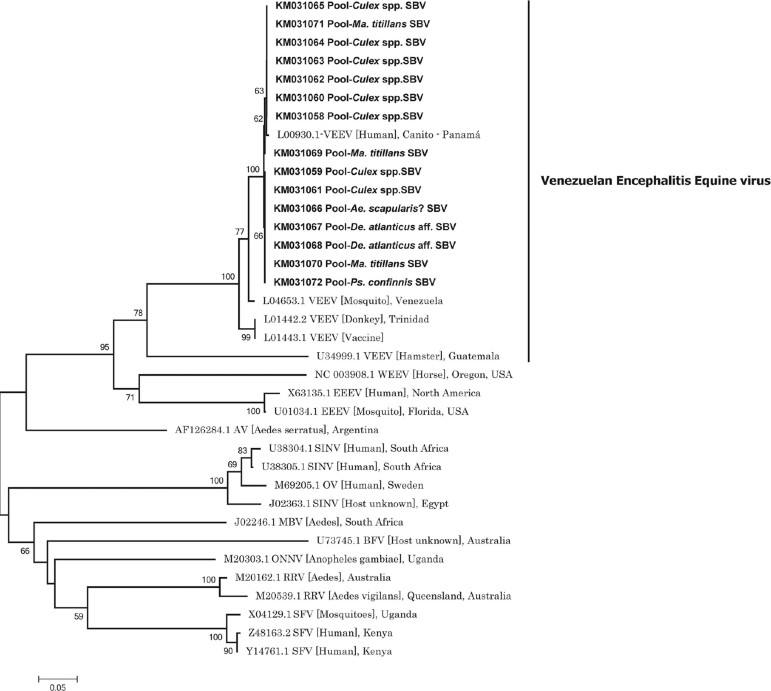
: neighbour-joining analysis used to identify *Alphavirus* sequences detected in mosquitoes (420 nt, nSP4). A dendrogram was constructed using representative sequences from the *Alphavirus* genus available in GenBank. The sequences detected were similar to representative Venezuelan equine encephalitis virus (VEEV) sequences. Abbreviations: WEEV (Western equine encephalitis virus); EEEV (Eastern equine encephalitis virus); AV (Aura virus); SINV (Sindbis virus); OV (Ockelbo virus); BFV (Barmah Forest virus); MBV (Middelburg virus); ONNV (o’nyong nyong virus); RRV (Ross River virus); and SFV (Semliki Forest virus). GenBank accession numbers are followed by the abbreviated names of viruses. The sequences of arboviruses detected in this study are presented with a GenBank accession number, followed by the mosquito species in which they were detected (in bold). SBV: San Bernardo del Viento, Córdoba (Colombia).

**Fig. 4:**
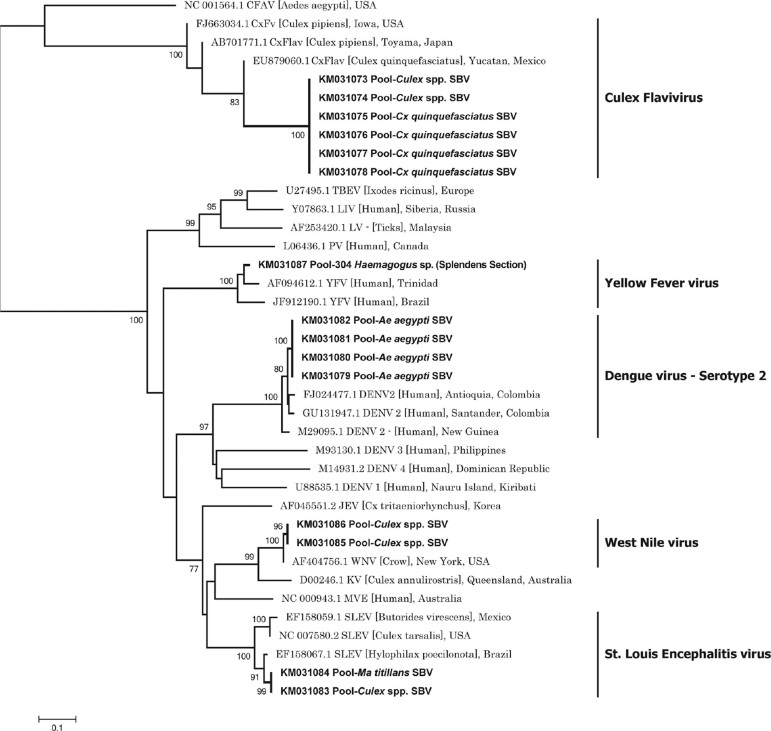
: neighbour-joining analysis used to identify *Flavivirus* sequences detected in mosquitoes (515 nt, NS5). A dendrogram was constructed using representative sequences of the *Flavivirus* genus available in GenBank. The sequences detected were similar representative sequences of dengue virus serotype 2 (DENV2), yellow fever virus (YFV), West Nile virus (WNV), St. Louis encephalitis virus (SLEV), and *Culex flavivirus* (CxFV). Abbreviations: PV (Powassan virus); LV (Langat virus); LIV (louping ill virus); TBEV (tick-borne encephalitis virus); KV (Kunjin virus); MVE (Murray Valley virus); JEV (Japanese encephalitis virus); CFAV (cell fusing agent virus). GenBank accession numbers are followed by the abbreviated names of viruses. The sequences of arboviruses detected in this study are presented with a GenBank accession number, followed by the mosquito species in which they were detected (in bold). SBV: San Bernardo del Viento, Córdoba (Colombia).

The alphaviruses detected in pools of *Culex* spp., *De. atlanticus* aff*.*, *Mansonia titillans*, *Psorophora confinnis*, and *Aedes scapularis* were all identified by their sequences as VEEV (GenBank accession numbers KM031058-KM031073) (Fig. 3). The flaviviruses were identified as DENV serotype 2 (DENV2) (*Ae. aegypti*), YFV (*Haemagogus* splendens), SLEV (*Ma. titillans*, *Culex* spp.), WNV (*Culex* spp.), and CxFV (*Culex* spp., *Cx. quinquefasciatus*) (Fig. 4) (GenBank accession numbers KM031074-KM031087). CxFV was the most commonly detected flavivirus (GenBank accession numbers KM031073-KM031078).

The MIR of SLEV, YFV, and WNV calculated for each morphospecies was low; the notable exceptions were CxFV in *Cx. quinquefasciatus* and DENV2 in *Ae. aegypti*. VEEV was detected in several mosquito species (Table).

VEEV was mostly detected in mosquitoes collected in mangrove forests or in neighbouring ecosystems (mangrove edge, 11 pools; rice crops in mangrove forest, six pools; and mangrove forest, five pools). SLEV, WNV, and YFV were detected only in mangroves. DENV2 was present in peridomestic sites, and CxFV was only detected in secondary forests.

## DISCUSSION

The presence of five arboviruses (genera *Alphavirus* and *Flavivirus*) indicates the risk for emergence or re-emergence of these pathogens in San Bernardo del Viento.

The only alphavirus detected was VEEV, which was found in *Culex* spp., *Ps. confinnis*, *De*. a*tlanticus* aff., *Ae. scapularis*, and *Ma. titillans* specimens. The frequency of VEEV detection and the number of mosquito morphospecies found infected with the virus may facilitate its transmission to reservoirs and possibly to humans (Ferro et al. 2003, 2015, Weaver et al. 2004). In Colombia, VEEV epidemics have been documented in various regions (Soriano-Lleras & Figueroa 1942, Sanmartin-Barbieri & Osorno-Mesa 1954, SanMartín et al. 1971, Rivas et al. 1995, Ferro et al. 2008, 2015), 2015), with the following mosquito species implicated in transmission: *Ma. venezuelensis*, *Ae. serratus*, *Aedes* (*Ochlerotatus*) spp., *Ae. fulvus*, and *Culex* (*Melanoconion*) spp. (Groot 1996, Weaver et al. 2004).

Many VEEV isolates have been obtained previously from the Magdalena valley (Ferro et al. 2003) and Norte de Santander (Ferro et al. 2008). In addition, *Culex* (*Melanoconion*) spp. infected with VEEV have been found in other localities in Colombia, such as Rionegro, Puerto Boyacá, and presumably Lozanía, Tolima; *Cx.* (*Melanoconion*) *aikenii* (syn. *Cx.* (*Melanoconion*) *ocossa* and *Cx.* (*Melanoconion*) *panocossa*) were determined to be responsible for an epidemic of VEEV in 1973 (Groot 1964). Other species that have been reported as VEEV infected include *Cx.* (*Melanoconion*) *vomerifer*, *Cx.* (*Melanoconion*) *pedroi*, and *Cx.* (*Melanoconion*) *adamesi* in the Monte San Miguel forest in the middle Magdalena valley (Ferro et al. 2008). Most strains of VEEV isolated from the *Culex* subgenus *Melanoconion* in Colombia have been categorized as enzootic subtype ID (Weaver et al. 1996, 2004). Our sequences grouped with VEEV subtype ID strain 3880, which was isolated from a human case in Canito, Panama (Brault et al. 2002). Parra-Henao and Suárez (2012) reported the presence of some *Melanoconion* species, including *Cx. (Mel.) erraticus*, *Cx. (Mel.) pedroi*, and *Cx. (Mel.) taeniopus*, that were the first mosquito species identified by both, morphology and DNA barcoding, in an area adjacent to San Bernardo del Viento (Hoyos-López et al. 2015a, b). However, Cytochrome Oxidase I or DNA-barcode fragment - COI sequences from specimens of the *Culex* subgenera *Melanoconion* and *Culex* did not allow molecular differentiation between these species, similar to that reported for species Cx. *coronator* in the subgenus *Culex* in Colombia (Rozo-Lopez & Mengual 2015, Hoyos-López et al. 2015d), *Cx. tarsalis* and *Cx. coronator* in Brazil and Argentina (Laurito et al. 2013), and *Cx. salinarius* in Brazil (Demari-Silva et al. 2011).

In our study, *Culex* spp., *Ps. confinnis*, *Ae. scapularis*, and *Ma. titillans* were found to be infected with VEEV. These species are likely enzootic vectors of the virus, as has been reported in various geographic regions of Colombia and in nearby countries (Groot 1964, Hastriter & Lawyer 1998); however, the local vector status of these four species is unknown. Our study is the first report of a natural VEEV infection in *De. atlanticus* aff. This morphospecies was associated with crab holes in mangroves forests that are near the beach and in coconut groves. The adults of this species are reported to have nocturnal or crepuscular habits, resting by day in the upper parts of crab holes (Belkin & Hogue 1959). Their biting activity may extend to humans (Adames 1971). However, many essential aspects of *De. atlanticus* aff. biology remain unknown. In the Guajira department, *De. atlanticus* aff. is the dominant mosquito species. It has been collected inside houses and is a vector of VEEV in this geographic area (Ferro et al. 2015).

SLEV and WNV are emerging flaviviruses that have been detected previously in humans, equines, and birds in the Colombian Caribbean region (Mattar et al. 2005a, b, 2011, Goéz-Rivillas et al. 2010, Osorio et al. 2012). Both viruses detected in this study were molecularly characterised based on the NS5 and envelope genes and were found to be closely related to genotypes associated with attenuated virulence (WNV Texas genotype) (Hoyos-López et al. 2015a) and little activity in human populations (SLEV genotype IV) (Hoyos-López et al. 2015b).

The CxFV detected in this study in pools of *Cx*. *quinquefasciatus* is the first evidence of this virus in Colombia, although it has been reported in the same species in other countries (Hoshino et al. 2007, Morales-Betoulle et al. 2008, Cook et al. 2009, Farfan-Ale et al. 2009, Kim et al. 2009, Huanyu et al. 2012, Machado et al. 2012). A strong ecological association between CxFV and WNV was observed in Chicago, USA (Newman et al. 2011), suggesting that super-infection with WNV during CxFV infection may interfere with secondary viral infection with a similar virus (Farfan-Ale et al. 2009). However, experimental infections of *Cx. quinquefasciatus* with WNV strain Guatemala and CxFV strain Izabal do not support this hypothesis, because prior infection with CxFV had no significant impact on WNV replication, infection, dissemination, or transmission by this mosquito (Kent et al. 2010). It is possible that natural infection with CxFV affects the vector competence of *Cx. quinquefasciatus* through vertical transmission and persistence in mosquito progeny (Bolling et al. 2012), influencing the ecology of viruses such as WNV and SLEV and even enhancing WNV transmission (Kent et al. 2010). Interestingly, *Cx. quinquefasciatus* was collected only in secondary forests and was absent from peridomestic areas. Possible explanations include the low density (approximately 30) of human households close to mangroves, saline water, and availability of few breeding sites (Forattini et al. 2002b, Correia et al. 2012). Potential breeding sites (many ponds and livestock watering devices) were found near the forest collection site.

In Colombia, vectors that participate in sylvatic YFV transmission include *Haemagogus equinus* and *Haemagogus janthinomys* (Morales 1968). Natural infection of *Haemagogus* sp*.* (Splendens section) with YFV may suggest an epidemiological risk for humans working in mangroves, as this species readily bites humans and is present in high densities in coastal mangrove forests (Arnell 1973). There have been previous reports of experimental and natural infection of YFV (Galindo et al. 1956, de Rodaniche et al. 1957) and SLEV in *Haemagogus* from Panamá (Kramer & Chandler 2001). In San Bernardo del Viento, cases of yellow fever have not been recorded; however, they are reported annually in the Caribbean region of the Cesar, Guajira, and Magdalena departments (Rojas-Álvarez 2008).

DENV2, and predominantly the American/Asian genotype (subtype IIIb), is frequently detected in patients in Colombia (Méndez et al. 2012). Although there are few cases in San Bernardo del Viento, with approximately 4-6 patients reported between 2011 and 2014 (Secretaría de Salud Municipal, San Bernardo del Viento, personal communication), asymptomatic human cases and clinical underreporting is likely common in other regions of Colombia (Méndez et al. 2006).

We report the first detection of flaviviruses and alphaviruses circulating in coastal mangroves and neighbouring ecosystems in Colombia, indicating a potential health risk to humans living or working in these zones. Interestingly, in the present study, the mangrove edge harboured the most positive pools of VEEV. Some authors have suggested an important role for the transitional zone between two adjacent ecological systems (e.g., mangroves and pastures) or “ecotones” in the transmission of emerging viruses and arboviruses (Estrada-Franco et al. 2004, Despommier et al. 2006). Mosquitoes that vector several viruses (i.e., Rift Valley fever, VEEV, YFV) have been found to be abundant in ecotones (Olival & Daszak 2005, Despommier et al. 2006), and they may have the potential to adapt and exploit new breeding sites in human-dominated ecosystems (Diallo et al. 2012). The presence of *Culex* subgenera *Melanoconion* and *Culex* may be important to the emergence and/or re-emergence of WNV, SLEV, and VEEV. Mangrove ecosystems, may be specifically acting as a source of emerging pathogens that infect nearby human populations (Holmes 2008). Furthermore, ecotones have been identified as potentially important for host seeking and host switching (Burg 2001, Méndez et al. 2001, Hoberg et al. 2002). Because of the high concentrations of reservoirs and potential hosts, jumps to alternative hosts or mosquitos with different levels of vector competence may be favoured (Barrera et al. 2002, Deardoff et al. 2011). A key factor is that RNA arboviruses have high rates of evolution that may facilitate the generation of progeny with variable fitness or virulence in alternative hosts (Holmes 2008, 2009, Hoyos et al. 2012).

All of these ecological and evolutionary factors may play a role in niche changes and consequent “jumps” of new pathogens to humans (Weaver 2005, Despommier et al. 2006, Holmes 2008, 2009, Hoyos et al. 2012). Therefore, it will be important to further study some aspects of arbovirus ecology in San Bernardo del Viento, such as the diversity of possible reservoirs, including migrant/resident birds, rodents, opossums, and bats. The wildlife commonly associated with mangrove forests around “La Balsa” include 296 species of birds (belonging to 61 families, of which 64 species are migratory) (Estela et al. 2005, 2010, Arzuza et al. 2008, Ruiz-Guerra et al. 2008), reptiles (Rojas & Sierra-Correa 2010), and mammals such as rodents (*Oligoryzomys* spp. and *Zygodontomys* spp.), bats (*Artibeus* spp., *Carollia* spp., and *Sturnira* spp.), sloths (*Bradypus* spp.), opossums (*Didelphis marsupialis*) (unpublished data), and primates (*Aotus lemurinus*, *Ateles geoffroyi*, *Cebus albifrons*, and *Saguinus oedipus*) (Rojas & Sierra-Correa 2010). Other factors such as the spatial distributions of potential reservoirs, arboviruses present in these communities, and evolutionary variants (e.g., low virulence) of detected arboviruses may explain the absence of encephalitis/haemorrhagic fever cases in humans (Mattar et al. 2005b, Weaver 2005, Hoyos-López et al. 2015a, b).

Arbovirus detection is just one aspect of vector incrimination, and more data are needed to identify potential vectors, define vector competence, and identify specific habitats, biting behaviour, and blood feeding patterns of possible vectors. All of these aspects should be considered in a context of global climate change, which is another factor that may contribute to the emergence of new diseases (Naicker 2011, Butler 2012) and may be particularly important in Colombia, which experiences the effects of climatological oscillations such as ENSO (Poveda et al. 2001, Singh 2013). These abiotic changes may contribute to an increase in the size of mosquito populations and in cases of vector borne diseases (Poveda et al. 2000). These perspectives may contribute to the development of more effective disease prevention and control strategies.
