# Supplementary material for: Molecular detection of flaviviruses and alphaviruses in mosquitoes (Diptera: Culicidae) from coastal ecosystems in the Colombian Caribbean
Source: Mem Inst Oswaldo Cruz. 2016 Oct 3;111(10):625–34. doi: 10.1590/0074-02760160096 (PMC5066328; doi:10.1590/0074-02760160096)
Supplement: Supplementary file 1 [file 0074-0276-mioc-0074-02760160096-suppl01.pdf]

 Mem Inst Oswaldo Cruz, Rio de Janeiro, Vol. *111*(10): 1-1, October 2016

TABLE

Primers and reverse-transcription-polymerase chain reaction (RT-PCR) (generic/nested) conditions for arboviral detection in target groups

Arboviruses group	Primers	Gene target	Conditions for RT-PCR	Reference
Alphavirus	Alpha1+ GAYGCITAYYTIGAYATGGTIGAIGG<br>Alpha1- KYTCYTCIGTRTGYTTIGTICCIGG	nSP4	1 cycle - 45 min/48°C<br>40 cycles: 30 sec/94°C, 1 min/52°C, 30 sec/68°C<br>1 cycle - 5 min/68°C	58
	Alpha2+ GIAAYTGYAAYGTIACICARATG<br>Alpha 2- GCRAAIARIGCIGCIGCYTYIGGICC		1 cycle - 2 min/94°C<br>40 cycles: 30 sec/94°C, 1 min/52°C, 15 sec/72°C<br>1 cycle - 5 min/72°C	
Flavivirus	Flavi1+ GAYYTIGGITGYGGIIGIGGIRGITGG<br>Flavi1- TCCCAICCIGCIRTRTCRTCIGC	NS5	1 cycle - 45 min/38°C<br>40 cycles: 30 sec/94°C, 1 min/47°C, 75 sec/68°C<br>1 cycle - 5 min/68°C	57
	Flavi2+ YGYRTIYAYAWCAYSATGGG<br>Flavi2- CCARTGITCYKYRTTIAIRAAICC		1 cycle - 2 min/94°C<br>40 cycles: 30 sec/94°C, 1 min/47°C, 15 sec/72°C<br>1 cycle - 5 min/72°C	

Supplementary data
